# *Trans* fatty acids in the Portuguese food market

**DOI:** 10.1016/j.foodcont.2015.12.010

**Published:** 2016-06

**Authors:** Nádia Costa, Rebeca Cruz, Pedro Graça, João Breda, Susana Casal

**Affiliations:** a LAQV@REQUIMTE, Faculty of Pharmacy, University of Porto, Rua de Jorge Viterbo Ferreira 228, 4050-313 Porto, Portugal; b Faculty of Nutrition and Food Sciences, University of Porto, Rua Dr. Roberto Frias, Porto 4200-465, Portugal; c Directorate General for Health (Direcção Geral de Saúde), Lisbon, Portugal; d Programme Manager Nutrition, Physical Activity and Obesity, Division of Noncommunicable Diseases and Life-course, WHO Regional Office for Europe, UN City, Marmorvej 51 |DK - 2100 Copenhagen, Denmark

**Keywords:** *Trans* fatty acids, Hydrogenated fat, Labelling, Safety and authenticity, Contaminants, Food chemistry

## Abstract

Consistent evidence exist on the harmful health effects of industrial *trans* fatty acids (TFA). In order to have accurate data on TFA intake and implement adequate measures to reduce their intake, each country should have updated estimates of TFA content in the diet. The objective of the present study was to provide data on the TFA content in food commercialized in the Portuguese market. The results on the TFA content of 268 samples acquired between October and December 2013 are reported. Samples were categorized as margarines and shortenings (n = 16), spreadable chocolate fats (n = 6), fried potatoes and chips (n = 25), industrial bakery (n = 4), breakfast cereals (n = 3), pastry products (n = 120), seasonings (n = 5), instant soups (n = 5), instant desserts (n = 6), chocolate snacks (n = 4), microwave popcorn (n = 4), cookies, biscuits and wafers (n = 53), and fast-food (n = 13), with butter (n = 4) included for comparison purposes. TFA were quantified by gas chromatography. Total TFA content in the fat ranged from 0.06% to 30.2% (average 1.9%), with the highest average values in the “biscuits, wafers and cookies” group (3.4% TFA), followed by the pastry group (2.0%). Fifty samples (19%) had TFA superior to 2% in the fat. These findings highlight there is still much need for improvement in terms of the TFA content in Portuguese foods, particularly in traditional pastry.

## Highlights

• T*rans* fatty acids content in Portuguese food is updated.
• Most food categories have very low TFA contents.
• Some fats used in local pastry have high TFA contents.
• Inaccurate fat designations might induce incorrect choices.

## 1 Introduction

There is consistent evidence of industrial *trans* fatty acids (TFA) adverse health effects, particularly on blood lipoprotein profiles, coronary heart disease, cancer and diabetes, while no reports are available on any beneficial health impact (Mensink and Katan, 1990, Ascherio, 2006, Booker and Mann, 2008, Mozaffarain et al., 2009, Teegala et al., 2009, Uauy et al., 2009, Brouwer et al., 2010, Bendsen et al., 2011). TFA elimination policies have shown huge potential to contribute to a reduction in mortality from NCDs and to reduce socioeconomic inequalities from CHD (Allen et al., 2015). Furthermore the potential for saving lives as a result of such policy has been highlighted elsewhere (Restrepo & Rieger, 2016).

Different approaches have been implemented to reduce TFA amounts in processed foods. Limitations on the content of industrialized TFA were implemented in some countries, as in Denmark since 2003 followed by Austria, Switzerland, Iceland, Norway, Hungary, Sweden (yet to be implemented) and more recently Latvia and Georgia, while others imposed mandatory labeling (USA, Brazil, etc.), or included recommendations for voluntary reduction by the industry, accompanied by nutritional recommendations and awareness programs on the adverse effects of TFA (Uauy et al., 2009, Downs et al., 2013). The WHO regional Office for Europe has recently produced a report highlighting the benefits and the importance of a trans-fat ban in Europe followed by a statement submitted by a group of civil society organizations and food industry operators supporting the idea of establishing a legal limit equivalent to a ban (WHO, 2015). Furthermore several member states of the EU have also written to the European Commission requesting it to explore a possible regulatory framework for the reduction of trans-fat.

Several surveys have been implemented in different countries, aiming to clarify the amounts of TFA ingested by different populations worldwide. The first and wider survey was the TRANSFAIR study, which took place between 1980 and 1996, and involved 14 European countries, Portugal included. This study estimated daily ingestions ranging from 1.2 g in Greece and Italy to 6.7 g in Iceland, with high variability between countries, food groups, and even genders (Hulshof et al., 1999). A decade later, and based on two surveys dated from 2005 to 2009, Stender et al., 2006, Stender et al., 2012 concluded that a general reduction was observed, but the consumption of TFA was still potentially high in some populations.

Regulation (EU) Nº, 1169/2011 of the European Parliament on the provision of food information to consumers determined that, by 13 December 2014, data on the presence of *trans* fats in the overall diet of the EU population should be known, in order to implement adequate measures for its reduction. Simultaneously, the European Food and Nutrition Action Plan 2015-2020, focuses on a reduction of diet related noncommunicable diseases, and includes a priority intervention on the elimination of *trans* fat, which should be limited to <1% of the daily energy intake, including those of natural origin (World Health Organization, 2003, World Health Organization, 2011). The recent Vienna declaration on Nutrition and NCDs, in the context of Health 2020 strengths the general commitment of all members to take decisive actions regarding healthier food, including a reduction of products with high TFA amounts, and implementation of common approaches to promote product reformulation (WHO, 2013).

In Portugal, only recommendations for voluntary reduction have been applied. However, while the TRANSFAIR study positioned Portugal within the countries with the lowest TFA contents in the nineties, the 2005 survey implemented by Stender et al. (2006) presented a worse panorama, with up to 43% of TFA in the fat of selected foods. Indeed, national data on table margarines (n = 40) sold in Portugal back in 1991 (Oliveira & Ferreira, 1993) showed a high prevalence of TFA in margarines (0.45%–14.2% in the fat). The two main margarines and shortenings industries in Portugal signed a commitment to reduce TFA in their products back in 1995 (FIPA, 2005) but, despite the visible reduction in 2002 (range 0.2–8.9%), particularly from the signatory industries, 80% of the samples were still prepared with hydrogenated or partially hydrogenated fats (Torres, Casal, & Oliveira, 2002). Latter, a survey of cookies and biscuits sold in Portugal (n = 100) was taken in 2006 (Casal et al., 2008), with a TFA range in the fat from 0.2 to 41.1% (average of 2.8%). Selected samples (n = 12) were reanalyzed in 2012 (Santos, Cruz, & Casal, 2015) with a clear reduction of TFA content, from an average of 5.35%–0.87%, but the general range was still high (0.11%–27.4%). No updated data on other food categories was found.

Based on the reduced and generally outdated information of TFA in Portuguese foods, and being this information mandatory for an accurate estimation of population exposure, the aim of this study was to determine the TFA content in Portuguese foods.

## 2 Materials and methods

### 2.1 Sample collection

In the absence of a representative national nutrition survey, with ingestion patterns and relative contributions to fat consumption, a preliminary desk review was performed on literature data, aiming to identify the food categories with potentially higher amounts of TFA from industrial origin in other countries (Hulshof et al., 1999, Chiara et al., 2003, Craig-Schmidt, 2006, Stender et al., 2006, Baylin et al., 2007, Ledoux et al., 2007, Griguol et al., 2007, Wagner et al., 2008, Richter et al., 2009, Fritsche et al., 2010, Remig et al., 2010, Cakmak et al., 2011; Hissanaga et al., 2012, Roe et al., 2013, Saunders et al., 2008). The following food categories were selected: bakery/breakfast cereals, biscuits/wafers/cookies, bouillon cubes, butter, chocolate snacks, chocolate spreads, fast food, instant desserts, instant soups, margarines/shortenings, pastry, popcorn, and potato chips/French fries. Butter was included only for comparison purposes, as it contains natural TFA.

Based on the aforementioned categories, a total of 268 samples were selectively purchased between October and December 2013. A worst case approach was implemented for each category, selecting samples whose labels indicated the presence of “partially hydrogenated fat” (PH) or “hydrogenated fat” (H) in the ingredients list, samples with insufficient or inexistent label information, and, when unavailable, samples with recognized market shares. Other fat sources found in the ingredients list comprised: vegetable fat (V), vegetable oils (O), mixtures of vegetable oils and fats (O/V), margarine (M), and butter (B). Most products were acquired in six main supermarkets chains in Portugal, being therefore representative of the nation acquisition pattern, and included samples produced in the European Union (EU) and non-EU samples. Due to the recognized importance of local traditional pastry, samples sold by small and privately owned shops were also included, from diverse geographical areas in Portugal.

### 2.2 Sample preparation

Each sample was carefully weight for dose or unit mass estimation. After being reduced to a homogeneous mass in a food processor, a representative sample portion was refrigerated (4 °C) and analyzed within two or three days. Most samples were analyzed as acquired, except for microwave popcorn and frozen puff sheets that were previously prepared according to manufacturer instructions.

### 2.3 Lipid extraction

Fat was extracted with a ternary mixture of cyclohexane, 2-propanol and aqueous NaCl solution (0.9%, w/v), enabling a fast and clean separation of the lipid phase (Smedes, 1999, Cruz et al., 2013, Santos et al., 2015). In brief, 500 mg of homogenized sample were extracted with 1.6 mL of 2-propanol and 2 mL of cyclohexane (analytical grade from Carl Roth GmBH, Germany) after addition of an internal standard for total fat estimation (glyceryl triundecanoate, Sigma–Aldrich, Spain). After vortex mixing and overnight maceration at 4 °C, aqueous NaCl was added (1%; 2.8 mL), thoroughly mixed and centrifuged at 5000 rpm for 5 min, and the upper phase was transferred to derivatization vials. After repeating extraction with further 2 mL of cyclohexane, the combined supernatants were evaporated under a stream of nitrogen at 60 °C.

### 2.4 Fatty acids analysis

Extracted lipids and internal standard were converted into their methyl esters (FAME) by cold alkaline derivatisation, following ISO 12966-2 (2011), using 2 M KOH in methanol. After a brief centrifugation (3000 rpm, 5 min) the supernatants were transferred to injection vials for the gas chromatograph auto-sampler.

**Fig. 1:**
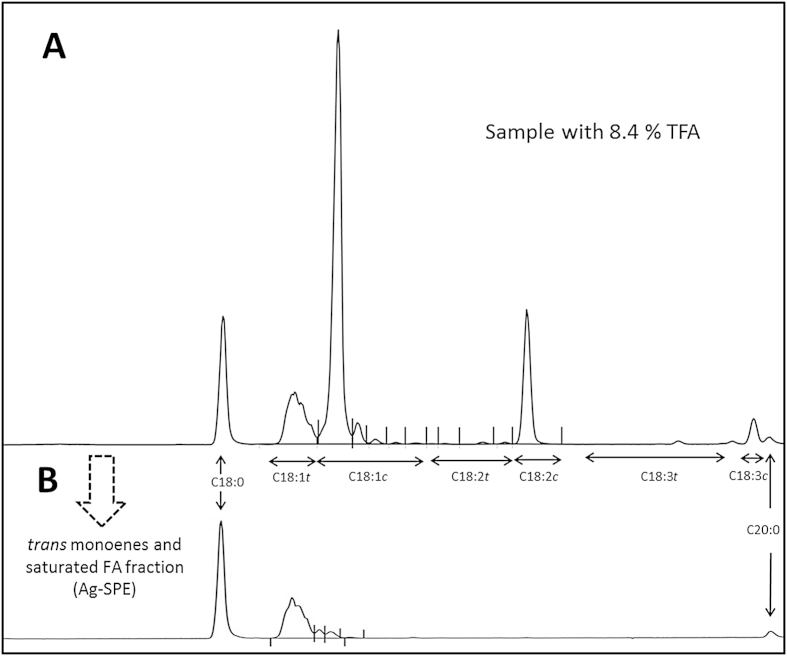
Chromatogram of a high TFA sample (A), with the detail of the *trans*-monoene fraction isolated by Ag-SPE (B).

The fatty acid composition was determined by gas chromatography on a Chrompack (CP 9001), equipped with a FAME CP-Select CB column (50 m × 0.25 mm x 0.2 μm; JW), with helium as carrier gas at 17 Psi, and a temperature gradient from 140 °C to 200 °C, in a total of 40 min. Injection port was at 250 °C, with a 1:100 split ratio, and the detector was at 270 °C. The fatty acids were identified by comparison with commercial standards form Supelco (Sigma, USA), and from Matreya (USA). A total of 52 fatty acids, with 8–24 carbon atoms, were quantified, including 11 *trans* isomers from C16:1 (n = 1), C18:1 (n = 4), C18:2 (n = 3), and 18:3 (n = 3). Following the recommendations of ISO 15304:2002, and for the purpose of this study (total TFA content estimation), total TFA in the fat is reported as the sum of all *trans* double-bond-containing fatty acid methyl esters, expressed as a mass fraction of all fatty acid methyl esters. The chromatographic conditions were adjusted to meet ISO 15304:2002 requirements for *trans* fatty acids separation. Isothermal elution as frequently described proved unsuccessful, while adequate separation was accomplished under the described temperature gradient. A chromatogram from a high TFA sample (8.4%) is presented in Fig. 1A.

Most reports on fatty acids contents use a relative percentage basis, extrapolated to food amounts from the total lipids content, quantified separately by gravimetry. Besides spending a huge amount of organic solvents and time, the extracted fat cannot be reused for accurate fatty acid quantification due to potential oxidation. Also, this “total fat” includes non-fatty acid components, which might take to overestimation of fatty acids on a 100 g of food basis. To overcome these issues, while enabling a faster and greener quantification, we have used cold organic extraction in the presence of a triglyceride as internal standard (gliceryl triundecanoate). Fat estimation achieved by this methodology was only used for fatty acid conversion into a food basis, and not as a true “total” fat. Therefore, while our total fat is certainly underestimated due to the sole inclusion of fatty acids glycerides, the estimation of the TFA in the food is more accurate. This methodology was previously validated by our team (Santos et al., 2015). A quantification limit of 0.01% in the fat was achieved under the present conditions.

Due to incomplete separation between individual *trans* fatty acid isomers in samples with high amounts of TFA (Fig. 1A), particularly within the *trans* octadecenoic cluster (C18:1), selected samples were further fractionated by solid phase (SPE) extraction, using Ag-SCX columns (Discovery Supelco, USA). Following manufacturer instructions, the SPE columns were pre-conditioned with acetone (4 mL) and equilibrated with hexane (4 mL). A FAME sample solution in hexane containing the equivalent to about 1 mg of FAMEs, with IS, was loaded into the column. Fraction 1, containing the saturated (including the internal standard) and all *trans* monoenes, was eluted with 6 mL hexane:acetone (96:4), while 4 mL hexane:acetone (90:10) were used to elute the *cis*-monoenes, *trans*–*trans* dienes and *cis/trans* conjugated fatty acids. Both fractions were evaporated under a stream of nitrogen, dissolved in hexane and re-chromatographed for validation of the *cis/trans* isomers identification and quantification. The *trans*-monoenes were quantified on the basis of the co-eluted internal standard, without significant differences from the values achieved without previous SPE separation, validating the efficiency of our chromatographic separation. A detail on the separation achieved for this fraction, plus the saturated ones, can be observed in Fig. 1B.

### 2.5 Statistical analysis

Dependent variables were studied using a Kruskal–Wallis test, when normal distribution of the residuals was not confirmed by Shapiro–Wilk's test, followed by Mann–Whitney's test if significant statistical differences were found. If normal distribution of the residuals was confirmed by Shapiro–Wilk's test, dependent variables were studied using Student's t test for independent samples. Statistical analyses were performed at a 5% significance level, using SPSS Software version 21.0 (IBM Corporation, New York, USA).

## 3 Results and discussion

### 3.1 Global TFA content

*Trans* fatty acids were detected in all samples, with total TFA in the fat ranging from 0.06% to 30.2%, and a global average of 1.87% (Table 1). From the 268 samples analyzed, and excluding butter, 50 samples (18%) had a TFA content superior to 2% in the fat, including six samples with more than 20% of TFA. The TFA content of the assembled food categories is detailed in Table 1, with the food categories ordered by their increasing average amount of TFA in the fat, ranging from 0.45% in chocolate spreads, to 3.42% in the biscuits, wafers and cookies group.

Total TFA content was inferior to 2% in all samples of the chocolate spreads group (n = 6), instant soups (n = 5), potato chips (n = 18), French fries (n = 7), bakery (n = 4), breakfast cereals (n = 3), chocolate snacks (n = 4), and microwave popcorn (n = 4).

The butter group, only analyzed for comparison purposes, had an average of 2.9%, within common values for this product (Kuhnt, Baehr, Rohrer, & Jahreis, 2011). The fast-food group (n = 13) had some samples slightly surpassing 2% of TFA in the fat (n = 3). However, it was associated with the presence of high amounts of cheese and meat (cheeseburgers), therefore also with TFA of natural ruminant origin. All the French Fries samples accompanying the fast food menus had less than 0.7% of total TFA in the fat.

A particular attention was given to the margarines and shortenings group (n = 16; average 0.83%), as these are used as ingredients and therefore amongst the main sources of TFA in processed foods. One sample slightly surpassed the 2% reference value (2.2%), while all the others were below. Within this group, 9 samples (56%) indicated the use of hydrogenated or partially hydrogenated fats, with a TFA average of 1.23%, with reduced statistical difference from the remaining margarine samples (0.59%; *p* = 0.083). Also within this groups, table margarines available to the consumers at supermarket chains (n = 9), as well as fats sold for semi-industrial use (n = 7) were included, but no statistical differences (p > 0.05) were observed between both groups (*p* = 0.299), with total TFA averages of 0.79% and 0.88%, respectively. However, when samples were grouped by origin, the Portuguese ones had apparently lower TFA average content (0.61%) than those made in the European Union (EU) (1.20%), but the high variability in the EU group (0.26–2.16%) decreased the potential statistical significance (*p* = 0.119). Following a reduction in the Portuguese margarines TFA content already visible in 2002, with an average of 2.5% (range 0.2–8.9%) (Torres et al., 2002), the TFA average in the present survey was reduced to 0.8% (range 0.2–2.16%), despite the declared use of hydrogenated fats in 40% of the samples. It might be the result of using a smaller proportion of hydrogenated fats in the blends or a more careful hydrogenation process, both contributing to a reduction in the TFA of the final product (Menaa, Menaa, Tréton, & Menaa, 2013).

Table 1 also details the type of TFA isomers quantified. As expected, *trans*-C18:1 fatty acids were more prevalent, except in the samples with lower total TFA contents, as chocolate spreads, potato chips, and instant soups, where linoleic TFA isomers, despite reduced, became more prevalent. Still, the *trans*-linoleic amounts were low, with an average of 0.35% in the fat (from <0.01 to 1.73). These values are in agreement with other authors who also detailed the isomers fractions, as Ansorena et al., 2013, Richter et al., 2009, or Alonso, Fraga, and Juárez (2000). *Trans*-linolenic fatty acids were always present in reduced amounts in our samples, with an average of 0.06% (<0.38% in the fat), also as stated by the previous authors.

Finally, when the TFA are expressed per 100 g of food, an average of 0.47 g was calculated, ranging from 0.01 g to 6.0 g/100 g (Table 1). The highest average amounts are found in the biscuits and cookies group, followed by pastry and margarines. These amounts cannot be directly associated with the recommended serving, as it varies greatly between food classes.

Globally, the worst Portuguese panorama, both as total TFA in the fat and per 100 g of food, was found on the “biscuits, wafers and cookies” (n = 53) and pastry (n = 120) groups, with some samples presenting particularly high amounts of TFA. The “biscuits, wafers and cookies” group had an average of 3.42% TFA in the fat, with values ranging from 0.21% to 30.2%, while the pastry group (n = 120) had an average of 1.96% TFA in the fat, ranging from 0.07% to 8.5%. It is interesting to highlight that the TFA amounts found in the pastry fat were significantly higher than those quantified in the margarines and shortenings analyzed (*p* = 0.018). It clearly demonstrates that the fat sources used for pastry production are from other sources than those available to consumers, probably acquired directly by sales representatives from foreign companies.

Still within the pastry group, the samples were further divided in two groups, puff and non-puff (Table 2), based on the knowledge that different margarines and shortening are sold for each purpose. The non-puff pastry included simple croissants, donuts, waffles, chocolate cakes, cupcakes and chocolate filled sweet breads (n = 30), while the puff pastry (n = 90) included french-type croissants, palmiers, several local puff specialties and frozen pastry puff-sheets. Identical mean values and ranges were found for the two groups, highlighting that the fats used are similar regarding their partially hydrogenated fats content, and that the major source of variability is probably the fat producer itself. However, when industrial pastry (n = 38) is compared with the local pastries (n = 82), statistically higher TFA (*p* = 0.017) were present in the local pastry group, with an average of 2.27% against 1.30% in the industrial ones. While confirming that the fat sources are different, it stresses the effective commitment of the food industries to reduce TFA on their products, a problem probably not even recognized by local producers. It also highlights for the “labelling” effect, as all the industrial samples were packaged and labeled at least for the ingredients, as mandatory, while local pastry was sold unpackaged.

The cookies group was also studied in detail (Table 2). Samples were grouped into 4 major types: simple/plain (n = 11), covered/filled (n = 23), wafers (n = 13) and puff based (n = 6). Significantly smaller TFA amounts were found in simple cookies (Marie-type) mainly composed of oils and vegetable fats mixtures (O/V). None mentioned the use of hydrogenated or partially hydrogenated fats. The highest average amounts of TFA were found in the cookies with cream (covered/filled), with an average of 5.5% of TFA in the fat. It indicates that the TFA are present mostly in the fats used for the filling creams, requiring an elevated proportion of solid fats, inevitably richer in saturated fat and/or TFA, technologically similar (Griguol et al., 2007, Stender et al., 2006). When these samples are compared on a mass basis, per 100 g (Table 2), significantly lower TFA amounts are present in the plain cookies (0.08 g/100 g), while all the other groups had more than 0.5 g/100 g on average. However, this data in the biscuits/wafers and cookies group is biased by a small group of non-EU samples (n = 6), five covered/filled and one wafer, all with more than 15% of TFA in the fat. These were all samples from Brazil and all contained this information in the nutritional label, as mandatory in that country since 2006 (ANVISA, 2003, section 1), with a clear indication of the TFA per dose. If these six samples are taken aside, the mean amounts are reduced to 1.0 g/100 g with a maximum of 9.1% in the fat, similar to the one achieved in the pastry group, and reducing the number of samples with amounts superior to 2% in the fat to only three. These results are consistent with a previous survey took in 2012 for Portuguese cookies, were the only samples with high amounts of TFA were from this origin (Santos et al., 2015), a situation that has not improved.

### 3.2 TFA content according to the type of fat labelled

As stated in the sampling section, this survey focused mainly on samples indicating the presence of hydrogenated or partially hydrogenated fat in the ingredients list. However, it also included several unlabeled samples (38%), particularly from pastry and fast food. One could expect that the more common source of TFA should be partially hydrogenated fats, where hydrogenation is taken only to a certain point, preserving unsaturation and granting more adequate technological properties, while producing TFA.

Excluding unlabeled samples, “hydrogenated fat” was more commonly mentioned (37%; n = 62) in the present survey, being the only fat source in 22 samples, including one instant soup, one popcorn sample, instant desserts (chocolate mousse and chantilly), industrial pastry and cookies. Partially hydrogenated fat was declared in fewer samples, representing 9.6% of the labels (n = 16), alone (breakfast cereals, bread, industrial pastry and chocolate snacks) or in mixtures with hydrogenated fat, vegetable fat and oils, etc. Vegetable fat was more frequent (67%), usually in combination with vegetable oils, hydrogenated fats or partially hydrogenated fats.

Generally, fat spreads, potato chips, soups, sauces and popcorn presented low amounts of TFA, being in accordance to the type of fat detailed in their label, mainly vegetable fats. In the category of bakery and breakfast cereals, three samples were labeled for partially hydrogenated fat but had low contents of TFA in the fat and therefore negligible per dose. Similarly, the chocolate snacks and desserts group had two samples which indicated partially hydrogenated fat but their amount of TFA in the fat was only of 0.5%. In the biscuits, wafers and cookies group (Table 2), 17 out of 23 samples of the covered/filled group and 8 out of 13 wafer samples were prepared with hydrogenated fat. The reference to partially hydrogenated fat was present in only two puff-cookies, both from Portugal, with 0.3% and 7.8% TFA in the fat. Globally, it appears that there is probably some misperception between hydrogenated, partially hydrogenated, and vegetable fats among food manufacturers and/or label translators.

Despite being unable to discuss the fat type used in unlabeled samples, based on the TFA contents achieved in the pastry group, partially hydrogenated fats are certainly present in high amounts. Information of the *trans* fat health effects and contents in food should be disseminated so that producers and consumers can act in a conscious way (Hissanaga et al., 2012). According to Remig et al. (2010) consumer education is very important, thus educational programs should be developed to improve the ability of consumers to identify the presence of hydrogenated fat in the ingredients list. Hydrogenated fats are cheaper than their technological counterparts and therefore potentially more presented in lower-cost food or brands. Despite not being exclusive, it highlights for the potential increased risk of low-income consumers.

### 3.3 Portugal TFA content in food in comparison with other countries

In comparison with international data, total TFA concentrations in this survey are in line with the ones found in many European countries where a self-regulatory approach is in place. Potato chips, French fries, popcorns, bakery, breakfast cereals, instant soups, and sauces, contained less than 2% TFA in the fat. In the European survey undertaken by Stender et al. (2006), where 542 samples foods from 26 countries were analysed, Portugal had up to 14% of TFA in popcorn and 4% in nuggets and French fries, representing a clear improvement. All popcorn samples analyzed in the present study specified the fat source, with three using solely native palm fat and one with fully hydrogenated palm kernel fat, corroborated by the low TFA quantified. However, a special concern still necessary in the pastry and cookies groups, where some samples with high TFA content are still found (up to 30.2% in the fat) side by side with others where a clear effort has been implemented by the industry.

Several reports from different countries showed similar situations, with some products containing total amount of TFA higher than 2% of fat. Richter et al. (2009), by analyzing different types of food products from the Swiss market, found great variations of TFA content (0.0–29.3% in a rapeseed fat). In that survey, the highest mean values were also observed in fine bakery products and snacks, cakes and biscuits, with means around 6% and 4%, respectively. In Germany, two recent studies also revealed high amounts of TFA in bakery products and confectioneries, with up to 27% in bakery products in the first (Fritsche et al., 2010), and up to 40% of TFA in the second (Kuhnt et al., 2011). These authors also concluded that unpackaged bakery products had higher amounts of TFA compared to packaged ones, as observed in the present study. Similar results were found outside Europe, namely for margarines and table spreads in New Zealand, with up to 14.5% TFA (Saunders et al., 2008) or in Turkey, where TFA content in cakes ranged from 0.0% to 5.1% in the fat (Cakmak et al., 2011). However, several improvements have already been recognized in some countries, as for the generally low TFA in Spanish industrial pastry, as published by Ansorena et al. (2013).

## 4 Conclusions

Based on the 268 samples analyzed, one can infer that TFA are still present in Portuguese food products. Two categories of foods are of major concern: pastry and cookies. Pastry products, particularly non-industrial unpackaged ones, have an elevated prevalence of samples with TFA superior to 2%, while the cookies group presented the higher TFA amounts. Regarding the fat-type used, hydrogenated fat was more prevalent than partially-hydrogenated fat but it might be a misinterpretation of the raw materials specifications.

The elevated prevalence of TFA in the pastry group, a highly available low-price food product in Portugal, with elevated consumption, requires measures to substitute the fats used, as already been achieved in most industrial pastry.

Additionally, there is also an evident need to help consumers interpreting the new food labels implemented by the EU Regulation (EU) Nº, 1169/2011, particularly regarding *trans* fat. As it is not declared in the nutritional label, the general notion that partially hydrogenated and hydrogenated fats should be avoided could be better widespread.

## Disclaimer

João Breda is a staff member of the WHO Regional Office for Europe. The author alone is responsible for the views expressed in this publication and they do not necessarily represent the decisions or the stated policy of WHO.

## Figures and Tables

**Table 1:** **-***Trans* fatty acids content (mean and range) of selected processed food products sold in the Portuguese market.

Food group	n	Total TFA *g/100g fat*	>2% n	*Trans*-C18:1	*Trans*-C18:2	*Trans*-C18:3	Total TFA *g/100g food*
*g/100g fat*		
Chocolate spreads	6	0.45 (0.14–0.86)	0	0.09 (nd - 0.13)	0.17 (nd - 0.57)	0.12 (nd - 0.32)	0.18 (0.07–0.32)
Instant soups	5	0.51 (0.34–0.77)	0	0.15 (0.09–0.29)	0.28 (0.25–0.32)	0.02 (nd - 0.04)	0.07 (0.02–0.18)
Potato chips and French fries^a^	25	0.62 (0.17–1.26)	0	0.14 (0.02–0.89)	0.37 (0.06–0.72)	0.05 (nd - 0.18)	0.18 (0.05–0.38)
Bakery and Breakfast cereals	7	0.71 (0.40–1.02)	0	0.26 (nd - 0.82)	0.25 (0.03–0.48)	0.09 (0.01–0.37)	0.03 (0.01–0.07)
Chocolate Snacks	4	0.83 (0.26–2.00)	0	0.60 (nd - 2.00)	0.18 (nd - 0.47)	0.01 (nd - 0.02)	0.32 (0.07–0.75)
Margarines and shortenings	16	0.83 (0.26–2.16)	1	0.35 (nd - 1.66)	0.34 (0.01–0.89)	0.09 (0.02–0.22)	0.56 (0.16–1.57)
Popcorn	4	0.87 (0.50–1.54)	0	0.40 (0.10–1.27)	0.41 (0.24–0.62)	0.03 (0.02–0.03)	0.11 (0.08–0.13)
Instant desserts^b^	6	0.95 (0.06–3.05)	1	0.95 (0.06–3.05)	nd	nd	0.19 (0.01–0.78)
Boillon cubes	5	1.10 (0.60–2.79)	1	0.59 (0.16–2.11)	0.39 (0.25–0.53)	0.08 (0.02–0.23)	0.28 (0.12–0.85)
Fast Food^c^	13	1.15 (0.38–3.07)	3	0.61 (0.05–2.10)	0.29 (0.11–0.50)	0.12 (0.05–0.25)	0.15 (0.06–0.40)
Pastry^d^	120	1.96 (0.07–8.47)	35	1.47 (0.01–7.94)	0.36 (nd - 0.69)	0.06 (nd - 0.26)	0.49 (0.01–2.51)
Biscuits, wafers and cookies	53	3.42 (0.21–30.2)	9	2.89 (0.01–28.65)	0.42 (nd - 1.73)	0.06 (nd - 0.38)	0.72 (0.02–6.02)
(Butter)	4	2.92 (2.07–3.58)	(4)	1.95 (1.16–2.53)	0.58 (0.51–0.65)	0.17 (0.16–0.17)	1.46 (1.14–2.00)
Total	268	1.87 (0.06–30.2)	50^e^	1.38 (nd - 28.6)	0.35 (nd - 1.73)	0.06 (nd - 0.38)	0.47 (0.01–6.02)

TFA: *trans* fatty acid; nd: not detected.

a Pre-packed potato chips and French fries (all frozen and pre-fried).

b Chocolate mousse and “chantilly.

c Nuggets, pizza, hamburgers and menus with French fries.

d Croissants, donuts, waffles, chocolate filled sweet-bread, chocolate cakes, cupcakes, Portuguese traditional pastry and frozen puff sheets.

e Without butter.

**Table 2:** **-***Trans* fatty acids content (mean and range), fat type and origin of pastry and cookies groups.

Group	Type	Total TFA			Fat type								Origin	
n	g/100 g fat	g/100 g food	V	O/V	O	H	PH	M	B	U	PT	EU
Pastry	Non-puff	30	1.91 (0.07–8.07)	0.44 (0.01–2.51)	14	3	2	12	3	0	3	6	16	14
Puff	91	1.94 (0.28–8.47)	0.51 (0.05–2.29)	8	2	2	4	6	1	0	69	82	9
Cookies	Plain	11	0.51 (0.21–0.89)	0.08 (0.02–0.14)^A^	6	3	0	1	0	2	1	7	4	0
Covered/filled	23	5.46 (0.22–30.1)	0.99 (0.06–6.02)^B^	11	1	1	17	0	1	0	8	9	6
Wafer	13	3.02 (0.24–22.9)	0.82 (0.06–5.25)^AB^	7	0	3	8	0	0	3	2	10	1
Puff	6	1.79 (0.32–7.84)	0.66 (0.10–3.01)^B^	1	3	1	3	2	0	0	4	2	0

TFA: *trans* fatty acids; V: vegetable fat; O/V: oil and vegetable fats; O: vegetable oil; H: hydrogenated fat; PH: partially hydrogenated fat; M: margarine; B: butter; U: unknown; PT: Portugal; EU: European Union. For the cookies group, different letters in the same column indicate significant statistical differences in the Mann–Whitney test (*p* < 0.05).
